# Sperm-Specific Glycolysis Enzyme Glyceraldehyde-3-Phosphate Dehydrogenase Regulated by Transcription Factor SOX10 to Promote Uveal Melanoma Tumorigenesis

**DOI:** 10.3389/fcell.2021.610683

**Published:** 2021-06-25

**Authors:** Xia Ding, Lihua Wang, Mingjiao Chen, Yue Wu, Shengfang Ge, Jin Li, Xianqun Fan, Ming Lin

**Affiliations:** ^1^Department of Ophthalmology, Ninth People’s Hospital, Shanghai Jiao Tong University School of Medicine, Shanghai, China; ^2^Shanghai Key Laboratory of Orbital Diseases and Ocular Oncology, Shanghai, China

**Keywords:** sperm-specific glyceraldehyde-3-phosphate dehydrogenase, glycolysis, uveal melanoma, SOX10, glycolytic enzyme

## Abstract

Melanoma cells exhibit increased aerobic glycolysis, which represents a major biochemical alteration associated with malignant transformation; thus, glycolytic enzymes could be exploited to selectively target cancer cells in cancer therapy. Sperm-specific glyceraldehyde-3-phosphate dehydrogenase (GAPDHS) switches glyceraldehyde-3-phosphate to 1,3-bisphosphoglycerate by coupling with the reduction of NAD+ to NADH. Here, we demonstrated that GAPDHS displays significantly higher expression in uveal melanoma (UM) than in normal controls. Functionally, the knockdown of GAPDHS in UM cell lines hindered glycolysis by decreasing glucose uptake, lactate production, adenosine triphosphate (ATP) generation, cell growth and proliferation; conversely, overexpression of GAPDHS promoted glycolysis, cell growth and proliferation. Furthermore, we identified that SOX10 knockdown reduced the activation of GAPDHS, leading to an attenuated malignant phenotype, and that SOX10 overexpression promoted the activation of GAPDHS, leading to an enhanced malignant phenotype. Mechanistically, SOX10 exerted its function by binding to the promoter of GAPDHS to regulate its expression. Importantly, SOX10 abrogation suppressed *in vivo* tumor growth and proliferation. Collectively, the results reveal that GAPDHS, which is regulated by SOX10, controls glycolysis and contributes to UM tumorigenesis, highlighting its potential as a therapeutic target.

## Materials and Methods

### Cell Lines

The human melanoma cell lines OCM1, OM431, and MUM2B were kindly provided by J. F. Marshall (Tumour Biology Laboratory, John Vane Science Centre, London, United Kingdom), and they were cultured in Dulbecco’s modified Eagle’s medium (DMEM; Gibco, Carlsbad, CA, United States) at 37°C mixed with 10% fetal bovine serum (FBS) (Gibco). The human normal retinal pigment epithelium cell line ARPE-19(RPE) was generously provided by the Department of Ophthalmology, Ruijin Hospital, Shanghai Jiao Tong University School of Medicine, China. ARPE-19 cells were maintained in DMEM/F12 medium mixed with 10% FBS. The human cutaneous melanocyte cell line PIG1 was a kind gift from Prof. Caroline Le Poole (Loyola University, Chicago, United States), and the culture media for all cells included penicillin (100 U/ml) and streptomycin (100 μg/ml). Cells in log phase growth were grown as monolayers in culture flasks at 37°C and 5% CO_2_.

### Quantitative Real-Time PCR

Total RNA was isolated from melanoma cells using TRIzol Reagent (Invitrogen, United States) and transcribed to complementary DNA (cDNA) using the TaKaRa PrimeScript RT reagent kit according to the manufacturer’s instructions. Quantitative real-time PCR (qPCR) was performed on an ABI system. GAPDHS and SOX10 mRNA levels were detected and normalized to that of the control gene 18S. The gene primers used are in Table 1.

**TABLE 1:** The sequence of primers used in our study.

**Genes**	**Forward primer**	**Reverse primer**
HK2	TTGACCAGGAGATTGACATGGG	CAACCGCATCAGGACCTCA
GPI	GGAGACCATCACGAATGCAGA	TAGACAGGGCAACAAAGTGCT
PFKL	GCTGGGCGGCACTATCATT	TCAGGTGCGAGTAGGTCCG
ALDOA	CAGGGACAAATGGCGAGACTA	GGGGTGTGTTCCCCAATCTT
TPI	ACTGCCTATATCGACTTCGCC	AAGCCCCATTAGTCACTTTGTAG
GAPDHS	TGTGGGCATCAATGGATTTGG	ACACCATGTATTCCGGGTCAAT
PGK1	GAACAAGGTTAAAGCCGAGCC	GTGGCAGATTGACTCCTACCA
PGAM1	GTGCAGAAGAGAGCGATCCG	CGGTTAGACCCCCATAGTGC
ENO1	AAAGCTGGTGCCGTTGAGAA	GGTTGTGGTAAACCTCTGCTC
PKM2	AAGGGTGTGAACCTTCCTGG	GCTCGACCCCAAACTTCAGA
GAPDH	AGGTCGGTGTGAACGGATTTG	TGTAGACCATGTAGTTGAGGTCA
18S	CGGCGACGACCCATTCGAAC	GAATCGAACCCTGATTCCCCGTC

*18s was used as a reference primer. HK2, hexokinase 2; GPI, glucose-6-phosphate isomerase; PFKL, phosphofructokinase liver type; ALDOA, aldolase A; TPI, triosephosphate isomerase; PGK1, phosphoglycerate kinase 1; GAPDH, glyceraldehyde-3-phosphate dehydrogenase; PGAM1, phosphoglycerate mutase 1; ENO1, enolase 1; PKM2, pyruvate kinase M2.*

### Western Blotting

Total protein was lysed for 30 min on ice by radioimmunoprecipitation assay (RIPA) lysis buffer (Beyotime Institute of Biology, Shanghai, China) and phenyl methane sulfonyl fluoride (100:1), and the concentrations were measured by the Bradford assay. Equal amounts of proteins from all of the samples were separated by 10% sodium dodecyl sulfate/polyacrylamide gel electrophoresis (SDS/PAGE) and then transferred to polyvinylidene difluoride (PVDF) membranes. Primary antibodies against GAPDHS (ProteinTech; 1:500) and SOX10 (Abcam; 1:1,000) were used to recognize the target proteins.

### Establishment of Stable Cells

The 293T cells were transfected with plasmids encoding GAPDHS and SOX10 or with negative control by Lipofectamine 2000 (Invitrogen). The virus supernatant after cell transfection for 48 h was gathered and added to OCM1 and OM431 cells in the presence of 8 μg/ml of polybrene (Sigma-Aldrich). Virus-infected cells were selected by 5 μg/ml of puromycin. GAPDHS and SOX10 shRNAs were commercially purchased from Sigma-Aldrich. Transduction was performed using the same procedure described above. The efficiency of knockdown or overexpression was detected by qPCR and western blotting.

### Measurement of Glucose Uptake, Lactate Production, and ATP Production

A glucose assay kit (BioVision), a lactate assay kit (Promega), and an ATP assay kit (Promega) were used according to the manufacturer’s instructions to measure glucose uptake and lactate and ATP production.

### Cell Counting and Colony Formation Assays

For cell counting, 2 × 10^3^ cells were seeded in 24-well plates. Cell numbers were counted at days 1, 2, 3, and 4 at the same time. For colony forming, cells were cultured in 6-well plates at 1,000 cells per well for approximately 2 weeks, and then colonies were fixed with 4% paraformaldehyde and stained with 0.5% crystal violet for 30 min. Photographs were taken, and the experiment was repeated three times.

### Tumor Growth *in vivo*

All animal studies were approved by the Ethics Committee of Shanghai Ninth People’s Hospital. For xenograft experiments, OCM1 cells stably expressing non-targeting control (NTC) and shSOX10 were infected with viruses expressing empty vector (EV) or GAPDHS. For the *in vivo* tumor assay, equal numbers of established stable cells were injected subcutaneously into BALB/c nude mice. Tumor volumes were recorded and calculated with the formula length × width × width/2 every 7 days. After 42 days, all tumors were isolated, and the size was measured. Next, the tissue samples from the mice were fixed and prepared for further immunofluorescence analysis.

### Chromatin Immunoprecipitation

Chromatin immunoprecipitation (ChIP) experiments were conducted according to the ChIP Assay Kit (Millipore) protocol. Cells were collected and lysed in ChIP lysis buffer and then sonicated to obtain DNA fragments. Antibodies targeting SOX10 and negative control IgG were added to pull down the DNA–protein–antibody complexes. After crosslinking reversal and purification, the samples were prepared for PCR. Primers of the GAPDHS promoter region for specific qRT-PCR were as follows: forward: 5′-GACATCGTCCTCACCAATGTCA-3′, reverse: 5′-CTGATCCCCTGGTCCCTAGATG-3′.

### Statistical Analysis

The data were presented as mean ± SE. Student’s *t*-test was used to calculate *P* values. ^∗^,*P* < 0.05 means significant difference.

## Introduction

Metabolic reprogramming is a hallmark of malignant transformation across all cancer types (Ratnikov et al., 2017), and hyperproliferation displayed by cancer cells is partly due to the involvement of aerobic glycolysis [the Warburg effect (Warburg, 1956)], which provides energy and foundation for the biosynthesis of macromolecules (Deberardinis et al., 2008; Lunt and Vander Heiden, 2011; Ratnikov et al., 2017) even under normoxic conditions, thus providing them with a growth advantage.

Melanoma cells exhibit the Warburg phenomenon and can adapt to use multiple fuels to support the malignant phenotype (Bettum et al., 2015). It is increasingly evident that oncogenic activation directly upregulates glycolytic enzymes (Rimpi and Nilsson, 2007) and/or activates them through transcription factors such as HIF1α and MYC (Parmenter et al., 2014). For instance, MYC upregulates glycolytic enzymes involved in glycolytic activity, including lactate dehydrogenase A (LDHA), which catalyzes the conversion of pyruvate to lactate, and hexokinase 2 (HK2), the first rate-limiting enzyme in the glycolytic pathway (Zeller et al., 2003). The net result of these changes is an increase in glucose uptake and glycolytic activity. Multiple lines of evidence have established that upregulation of a series of metabolic enzymes, such as HK2, GAPDH, LDH, PGAM1, PKM2, and PDK, is linked to malignant growth (Hugo-Wissemann et al., 1991; Mikuriya et al., 2007; Yeh et al., 2008), and interfering with the process of glycolysis has been proposed as an effective way to control tumor growth. In addition to these enzymes, it is worth noting that other sperm-specific isoforms of glycolytic enzymes (Welch et al., 2000) are involved in glycolysis in various cancers, and not all the catalytic and regulatory parameters are the same as the somatic isoenzymes. Essentially, sperm-specific lactate dehydrogenase C (LDHC) is a glycolytic enzyme that was revealed to cause constitutive activation of the anaerobic pathway in cancers (Koslowski et al., 2002). Sperm-specific glyceraldehyde-3-phosphate dehydrogenase (GAPDHS) is an isoform of somatic GAPDH, and it has been suggested to be a key regulator of glycolysis in spermatogenic cells and the target of environmental compounds that disrupt male fertility (Liu et al., 2013). A previous analysis showed that GAPDHS mRNA expression is enhanced in some melanoma cell lines compared with controls (Sevostyanova et al., 2012), and it has recently been correlated with an adverse clinical outcome in stage III–IV melanoma (Falkenius et al., 2013). The main function of these enzymes is to provide energy for the contractive elements of the principal part of the sperm flagellum to make the sperm move (Sevostyanova et al., 2012). However, there is limited documentation of the functions of GAPDHS in tumors, and whether GAPDHS modulates glycolysis and tumorigenesis in uveal melanoma (UM) remains to be investigated.

Sry (sex determining region Y)-related HMG box 10 (SOX10) is a high-mobility group (HMG) transcription factor characterized by a conserved HMG domain that was originally thought to exert its effects on transcription due to its ability to bend DNA (Werner and Burley, 1997); this transcription factor plays a crucial role in the specification, migration, and survival of all non-ectomesenchymal neural crest derivatives, including melanocytes (Wan et al., 2011). While the majority of Sox proteins function as architectural transcription factors, some members of the Sox family may also promote glycolysis. For instance, SOX4 regulates glycolytic metabolism through the AKT signaling pathway (Dai et al., 2017), and SOX2-driven OXPHOS reprogramming occurs via HIF1α pathway disruption (Andreucci et al., 2018) in melanoma. SOX10 generally cooperates with other transcriptional regulators to synergistically activate transcription, binding to AT-rich motifs in the minor groove of DNA (Marathe et al., 2017). Microphthalmia-associated transcription factor (MITF) is found to promote expression of metabolic genes in melanoma (Ratnikov et al., 2017) and also has a role alongside SOX10 in regulating target genes (Hoek et al., 2008). SOX10 is specifically upregulated in melanoma tissues compared with control tissues according to the Gene Expression Profiling Interactive Analysis (GEPIA) database; in addition, it promotes both melanoma initiation and progression (Shakhova et al., 2012, 2015) and serves as a biomarker for the detection of UM (Alghamdi et al., 2015). Thus, it is worthwhile to investigate whether SOX10 plays an important role in the Warburg effect and modulates glycolytic enzymes as a transcriptional factor.

In this study, we demonstrate that GAPDHS is significantly elevated in UM and functions in the control of glycolysis and promotes colony formation *in vitro* and tumor formation *in vivo*. Mechanistically, we discovered that SOX10 regulates the expression of GAPDHS to modulate metabolic reprogramming and ultimately UM tumorigenesis.

## Results

### GAPDHS Is Highly Expressed in Melanoma Cells

**FIGURE 1:**
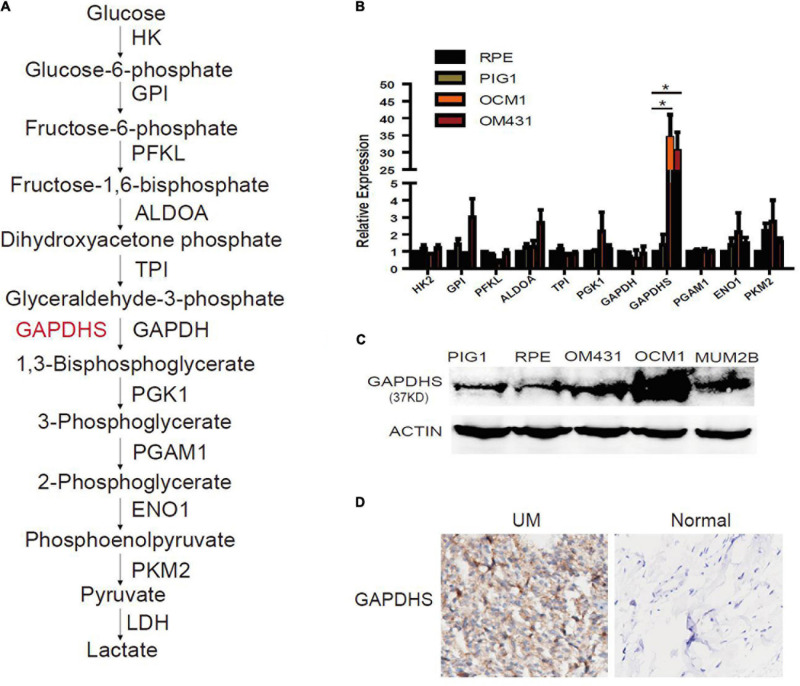
Aberrant expression of glyceraldehyde-3-phosphate dehydrogenase (GAPDHS) in melanoma. **(A)** Glycolysis enzymes involved in the glycolytic pathway. HK, hexokinase; GPI, glucose-6-phosphate isomerase; PFKL, phosphofructokinase liver type; ALDOA, aldolase A; TPI, triosephosphate isomerase; PGK1, phosphoglycerate kinase 1; GAPDH, glyceraldehyde-3-phosphate dehydrogenase; PGAM1, phosphoglycerate mutase 1; ENO1, enolase 1; PKM2, pyruvate kinase M2. **(B)** Glycolysis enzyme expression was determined by real-time qPCR in melanoma cell lines, and values are given as the mean ± SE. HK2, hexokinase 2; GPI, glucose-6-phosphate isomerase; PFKL, phosphofructokinase liver type; ALDOA, aldolase A; TPI, triosephosphate isomerase; PGK1, phosphoglycerate kinase 1; GAPDH, glyceraldehyde-3-phosphate dehydrogenase; PGAM1, phosphoglycerate mutase 1; ENO1, enolase 1; PKM2, pyruvate kinase M2. ^∗^*P* < 0.05 compared with the normal control group. ARPE-19 and PIG1 cells were used as normal controls. **(C)** Protein levels of GAPDHS were determined by western blot in OCM1 and OM431 cells. Actin served as the loading control. ARPE-19 and PIG1 cells were used as normal controls. **(D)** GAPDHS expression in 10 uveal melanoma tissues and 10 normal tissues by immunohistochemistry (IHC).

To identify the glycolytic enzyme that is responsible for glycolysis in UM, real-time PCR was performed in UM cells to detect the expression of glycolytic enzymes (Figure 1A). Notably, we observed that GAPDHS mRNA was highly expressed in UM cells compared with the normal cell lines ARPE-19 and PIG1 (Figure 1B), which is consistent with a previous report that GAPDHS expression is significantly higher in human melanoma cell lines than in normal controls (Sevostyanova et al., 2012). Further validation by western blotting using the two classic UM cell lines OCM1 and OM431 also revealed elevated GAPDHS expression (Figure 1C). We identified GAPDHS as a 37-kDa protein by western blot analysis, which is consistent with the results of a previous study (Sevostyanova et al., 2012) in which GAPDHS was reported to be a 37-kDa protein without an N-terminal domain that attaches the enzyme to the cytoskeleton of the sperm flagellum. Moreover, immunohistochemistry (IHC) showed that GAPDHS is significantly elevated in UM tissues compared with normal controls (Figure 1D). Thus, these data demonstrate that GAPDHS mRNA and protein are aberrantly highly expressed in UM tissues and cells, indicating that GAPDHS may play a vital role in UM progression, which makes it an attractive candidate for further investigation.

### GAPDHS Is Involved in Regulating the Warburg Effect in UM Cells

**FIGURE 2:**
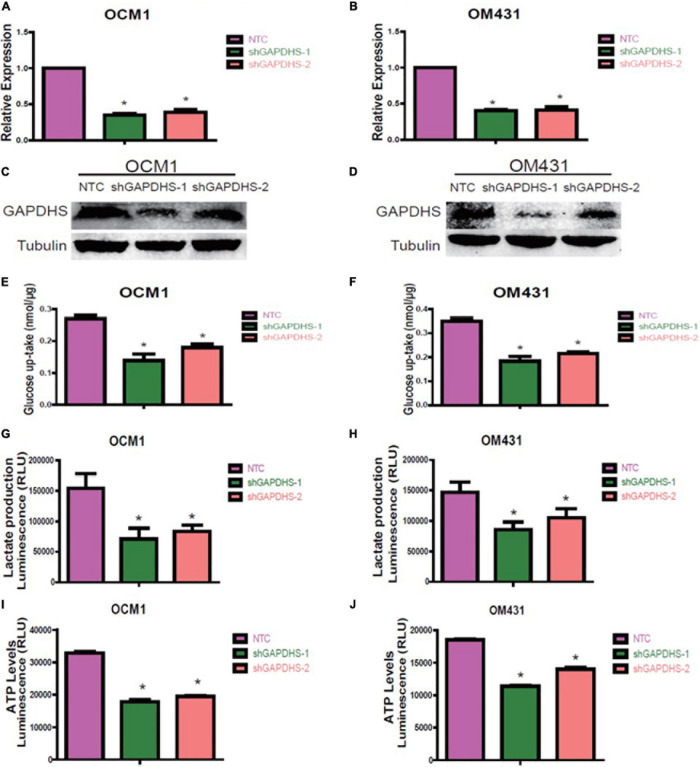
Glyceraldehyde-3-phosphate dehydrogenase (GAPDHS) knockdown inhibits glycolysis in melanoma cell lines. **(A,B)** Real-time PCR demonstrated that two shRNAs (shGAPDHS-1 and shGAPDHS-2) could silence GAPDHS expression in OCM1 and OM431 cells. Non-targeting control (NTC) indicates cells expressing NTC. *P < 0.05 compared with the NTC group. **(C,D)** Western blot verified that shGAPDHS-1 and shGAPDHS-2 efficiently silenced GAPDHS at the protein expression level in OCM1 and OM431 cells. Tubulin served as the loading control. **(E–J)** Glucose uptake **(E,F)**, lactate production **(G,H)**, and ATP levels **(I,J)** were measured in OCM1 and OM431 cells stably expressing NTC and two shRNAs of GAPDHS. Data are presented as the mean ± SE. *P < 0.05 compared with the NTC group.

**FIGURE 3:**
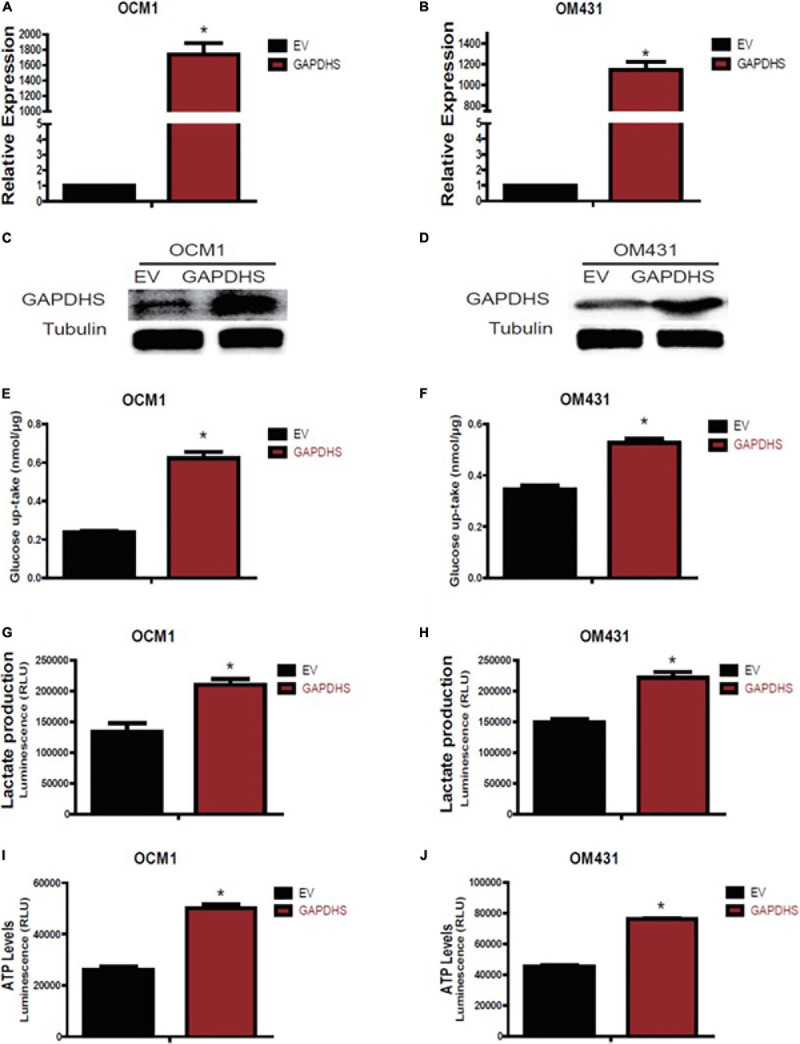
Glyceraldehyde-3-phosphate dehydrogenase (GAPDHS) overexpression promotes glycolysis in melanoma cell lines. **(A,B)** GAPDHS mRNA levels were determined by real-time PCR in OCM1 and OM431 cells stably overexpressing empty vector (EV) or GAPDHS. **(C,D)** GAPDHS protein levels were determined by western blotting in OCM1 and OM431 cells stably overexpressing EV or GAPDHS. Tubulin served as the loading control. **(E–J)** Glucose uptake **(E,F)**, lactate production **(G,H)**, and ATP levels **(I,J)** were measured in OCM1 and OM431 cells stably overexpressing EV or GAPDHS. Data are presented as the mean ± SE. *P < 0.05 compared with the EV group.

As multiple lines of evidence suggest that GAPDHS is a key regulator of glycolysis in spermatogenic cells (Eddy et al., 2003; Liu et al., 2013) and facilitates glycolysis (Sevostyanova et al., 2012) in melanoma cells, we then investigated whether the highly expressed GAPDHS regulates glucose metabolism in UM. To further determine the glycolytic functions of GAPDHS in UM *in vitro*, we established shRNAs to knock down GAPDHS expression. To control for non-specific effects, two different shRNAs were used in the OCM1 and OM431 cell lines, while a non-targeting shRNA was used as a control. Both shRNAs targeting GAPDHS worked efficiently, as confirmed by qRT-PCR and western blotting, compared with the NTC group (Figures 2A–D). Then, we found that the metabolic parameters including cellular glucose uptake, lactate production, and cellular ATP levels were significantly decreased in OCM1 and OM431 cells after GAPDHS knockdown (Figures 2E–J). Furthermore, GAPDHS overexpression cell lines were generated by plasmid transfection, and the efficiency was confirmed (Figures 3A–D). As expected, we observed that cellular glucose uptake, lactate production, and cellular ATP levels were markedly increased in the stable GAPDHS-overexpressing group compared with the EV group (Figures 3E–J), indicating that GAPDHS upregulation can accelerate glucose metabolism by increasing glucose uptake, lactate production, and cellular ATP levels. Taken together, our results from both weakened expression and forced expression studies suggested that GAPDHS facilitates aerobic glycolysis or the Warburg effect in UM cells.

### GAPDHS Promotes the Proliferation of UM Cells

**FIGURE 4:**
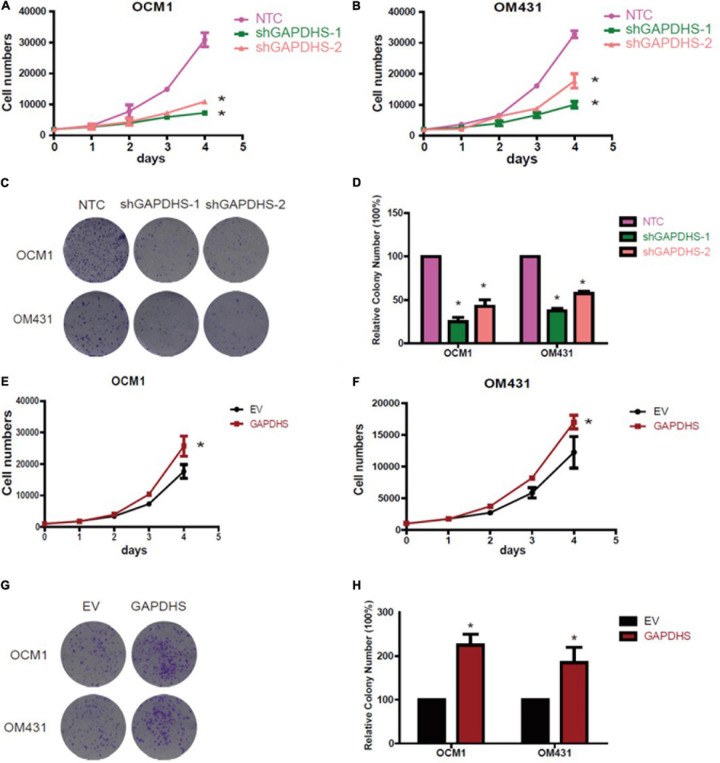
Glyceraldehyde-3-phosphate dehydrogenase (GAPDHS) promotes growth and proliferation *in vitro*. **(A,B)** Cell growth of melanoma cells stably expressing non-targeting control (NTC) or two shRNAs (shGAPDHS-1 and shGAPDHS-2) of GAPDHS. **(C,D)** Colony formation assay to assess the tumor colony forming ability of OCM1 and OM431 cells expressing Non-targeting control (NTC) and two shRNAs (shGAPDHS-1 and shGAPDHS-2) of GAPDHS. **(E,F)** Cell growth of melanoma cells stably overexpressing control (EV) or GAPDHS. **(G,H)** Colony formation assay to assess the tumor colony forming ability of OCM1 and OM431 cells overexpressing control (EV) or GAPDHS.

Since our results clearly demonstrated that GAPDHS regulates aerobic glycolysis in UM, we further explored whether GAPDHS is critical for cancer proliferation. To further determine the tumorigenic activity of GAPDHS in UM *in vitro*, we evaluated the effect on growth, and the results of the cell counting assay showed that the growth rate was inhibited after GAPDHS knockdown (Figures 4A,B). In addition, we assessed the role of GAPDHS in tumor formation with a colony-forming assay. Consistently, we recognized that GAPDHS silencing with shRNAs significantly decreased the number of visible colonies compared with that in the control group (Figures 4C,D). Furthermore, cell growth analysis revealed that forced expression of GAPDHS increased the growth rate (Figures 4E,F). Moreover, the stable GAPDHS-overexpressing group exhibited a significantly increased number of visible colonies compared with that in the EV-expressing control group (Figures 4G,H). In addition, we observed that GAPDHS knockdown caused cell cycle arrest at the G2/M phase (Supplementary Figure 1). Collectively, these findings demonstrated that GAPDHS has a pro-proliferative effect, indicating that GAPDHS may be involved in the malignant progression of UM.

### GAPDHS Is a Direct Target of SOX10

**FIGURE 5:**
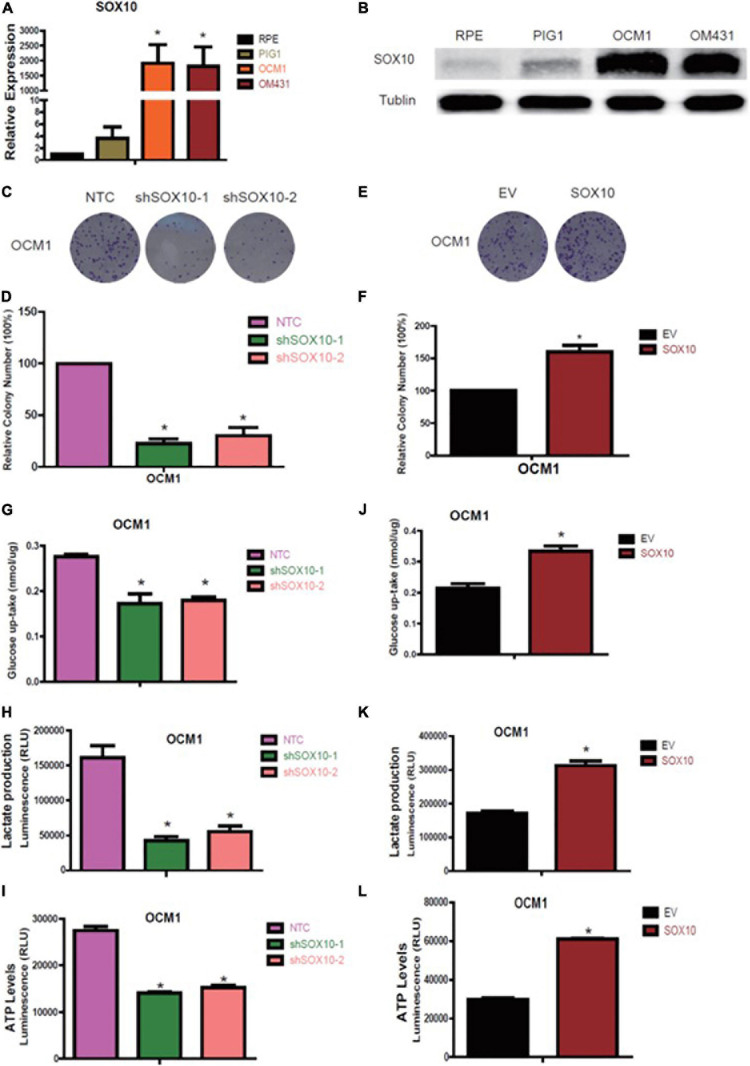
SOX10 promotes proliferation and glycolysis in melanoma cell lines. **(A,B)** RT-qPCR and western blot analysis of SOX10 expression in OCM1 and OM431 cells. **(C,D)** Colony formation assay to assess the tumor colony forming ability of OCM1. Non-targeting control (NTC) indicates cells expressing NTC, and shSOX10-1 and shSOX10-2 indicates cells harboring shRNAs of SOX10. **(E,F)** Colony formation assay to assess the tumor colony forming ability of OCM1 cells overexpressing empty vector (EV) or SOX10. **(G–I)** Glucose uptake **(G)**, lactate production **(H)**, and cellular ATP levels **(I)** were measured in OCM1 cells stably expressing NTC or two shRNAs (shSOX10-1 and shSOX10-2) of SOX10. Data are presented as the mean ± SE. *P < 0.05 compared with the NTC group. **(J–L)** Glucose uptake **(J)**, lactate production **(K)**, and cellular ATP levels **(L)** were measured in OCM1 cells stably overexpressing empty vector (EV) or SOX10. Data are presented as the mean ± SE. *P < 0.05 compared with the EV group.

Preliminary results have shown that glycolytic enzymes highly correlated with tumor prognosis may be regulated by tumorigenic factors or may regulate tumorigenesis in parallel. To determine the factors coordinating the variations in UM, we analyzed the potential transcription factors that might interact with the GAPDHS regulatory region to regulate GAPDHS expression. Among the multiple potential factors analyzed according to the RNA-seq for UM, we observed that SOX10 was markedly increased in OCM1 and OM431 cell lines compared with normal control cells by real-time PCR and western blot analysis (Figures 5A,B).

Hence, we subsequently established OCM1 cells with SOX10 loss of function with two different shRNAs and gain of function followed by puromycin selection to detect the functional role of SOX10 in UM and detected that shSOX10 markedly reduced the number of visible colonies compared with that in NTC cells using the colony formation assay (Figures 5C,D); the number of visible colonies increased when SOX10 was overexpressed (Figures 5E,F). In addition, we also recognized that knockdown of SOX10 significantly decreased glucose uptake and lactate and ATP production levels (Figures 5G–I), while these metabolic parameters markedly increased with forced expression of SOX10 (Figures 5J–L). Based on these findings, we confirmed that SOX10 promotes UM glycolysis and proliferation.

**FIGURE 6:**
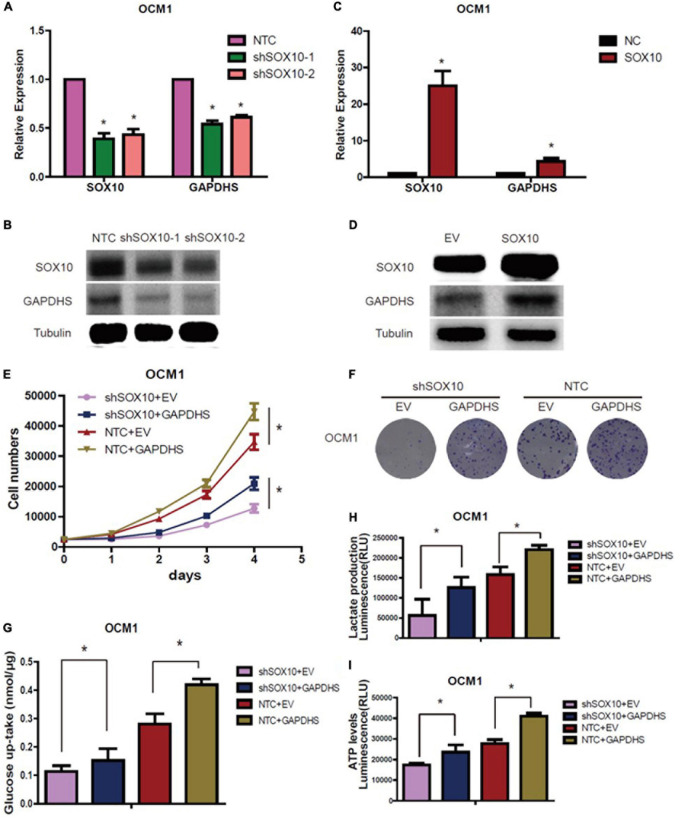
GAPDHS is a direct target of SOX10 *in vitro*. **(A,B)** GAPDHS expression was measured by real-time PCR and western blotting in OCM1 cells expressing non-targeting control (NTC) or two shRNAs (shSOX10-1 and shSOX10-2) of SOX10. Tubulin served as the loading control. **(C,D)** GAPDHS expression was measured by real-time PCR and western blotting in OCM1 cells overexpressing control (EV) or SOX10. Tubulin served as the loading control. **(E–I)** OCM1 cells stably expressing NTC or shSOX10 were infected with viruses overexpressing GAPDHS, followed by measurement of cell growth **(E)**, tumor colony forming ability **(F)**, cellular glucose uptake **(G)**, lactate production **(H)**, and ATP levels **(I)**. Data are presented as the mean ± SE. *P < 0.05.

Quantitative real-time PCR and western blot analysis results revealed that GAPDHS expression was markedly diminished when SOX10 was knocked down (Figures 6A,B), and it was significantly increased when SOX10 was overexpressed (Figures 6C,D). These data indicate that GAPDHS might be a target gene of SOX10. However, whether SOX10 exerts its function via GAPDHS, which is associated with glycolysis and proliferation, needs to be further elucidated. Then, we transfected a GAPDHS-overexpressing plasmid into OCM1 cells expressing shSOX10 to further assess whether the overexpression of GAPDHS could reverse the inhibitory effect of SOX10 knockdown on cell proliferation and the Warburg effect in UM cells. We found that the compromised oncogenic potential in the SOX10 downregulation group was partly countervailed by the overexpression of GAPDHS (Figures 6E,F), and glucose uptake, lactate production and ATP levels were obviously restored (Figures 6G–I). These results confirmed that GAPDHS serves as a functional target gene in SOX10-mediated tumor proliferation and glycolysis in UM. Similarly, we further explored whether SOX10 directly promotes GAPDHS expression and whether GAPDHS is critical for SOX10-regulated cancer proliferation *in vivo*. First, we overexpressed GAPDHS in OCM1 NTC and stable SOX10 knockdown cell lines and subcutaneously injected four cell lines (shSOX10 + EV, shSOX10 + GAPDHS, NTC + EV, and NTC + GAPDHS) into the right flank of nude mice. We then measured the size of the resultant tumors every 7 days for 42 days.

**FIGURE 7:**
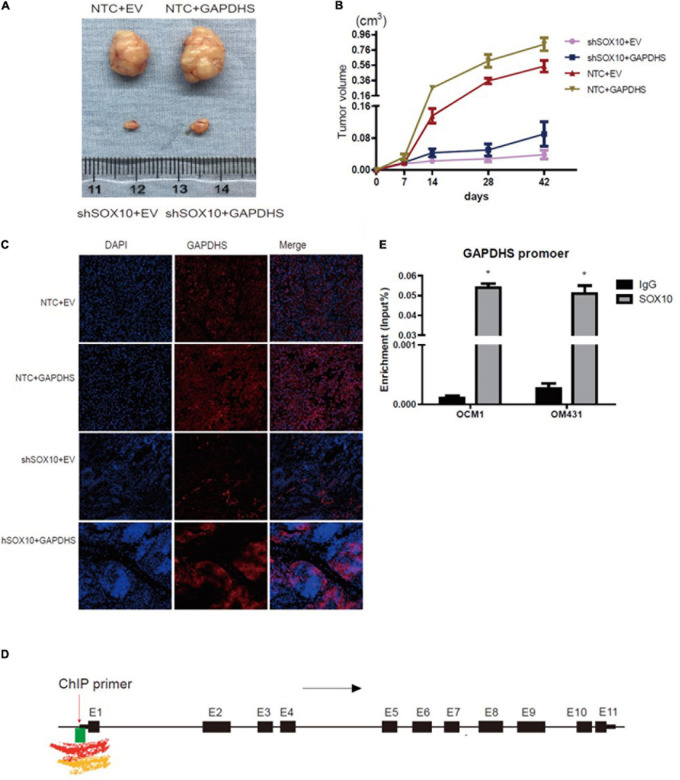
The mechanism of glyceraldehyde-3-phosphate dehydrogenase (GAPDHS) regulated by SOX10. **(A–C)** SOX10 regulates GAPDHS expression in an *in vivo* xenograft model assay (*n* = 6 for each group). **(A)** Tumor sizes on the 42nd day were compared at the end of the experiment. **(B)** Tumor growth curves were measured starting at 7 days after inoculation. **(C)** Representative immunohistochemistry (IHC) analysis of GAPDHS protein expression in tissues from nude mice. **(D)** Overview of H3K4me1, H3K4me3, and CpG island binding sites in the regulatory region of GAPDHS from the UCSC database. Black arrow, transcription direction; red arrow, chromatin immunoprecipitation (ChIP) primer used in this study, located upstream of the first exon of GAPDHS; black box, exons of GAPDHS; E1 through E11, number of exons; green box, CpG island enrichment around the first exon; red waveform, Encode Enhancer- and Promoter-Associated Histone Mark (H3K4me1) enrichment around the first exon; yellow waveform, Encode Promoter-Associated Histone Mark (H3K4me3) enrichment around the first exon. **(E)** ChIP analysis of OCM1 and OM431 cell lysates showing SOX10 recruitment to the GAPDHS promoter region. ^∗^*P* < 0.05.

Consistent with our previous *in vitro* results, the SOX10 knockdown group (shSOX10 + EV) in the OCM1 cell line exhibited significantly slower tumor growth than the control group (NTC + EV). Moreover, the size of tumors derived from OCM1 cells stably overexpressing GAPDHS (NTC + GAPDHS) was significantly enhanced compared with that size of those derived from the control group (NTC + EV). Importantly, we also observed that tumor growth increased in the GAPDHS overexpression group compared with that in the SOX10 knockdown group, and the overexpression of GAPDHS partly rescued the impairment of tumor growth by shSOX10 *in vivo* (Figures 7A,B). These results from *in vitro* and *in vivo* experiments suggest that SOX10 modulates the tumorigenesis of UM by regulating GAPDHS expression.

Furthermore, we detected the expression of SOX10 and GAPDHS in tissue samples from the mice through fluorescence staining, and the results confirmed the overexpression of GAPDHS and the downregulation of SOX10 with shRNAs. An analysis of xenografted tumor tissues from the mice revealed that tumors derived from SOX10 knockdown cells (shSOX10 + EV) had lower expression of GAPDHS than those derived from the control group (NTC + EV). As expected, we eventually confirmed that GAPDHS could partially offset the inhibitory effect of shSOX10 on melanoma growth (Figure 7C). These data indicated that SOX10 significantly promoted tumor growth via GAPDHS and further established that the tumorigenic effects of SOX10 heavily relied on GAPDHS expression, extending the role of GAPDHS as a master regulator of UM glucose metabolism.

### SOX10 Modulates GAPDHS Expression by Binding to the Promoter

According to the above results, to elucidate the mechanisms underlying the regulation of GAPDHS expression by SOX10 in UM cells, we speculated whether SOX10 might represent a putative GAPDHS regulatory region binding factor and whether its modulation of the Warburg effect might depend on the promotion of the transcriptional activity of GAPDHS. To confirm our hypothesis, we performed a bioinformatic analysis for the presence of putative SOX10-binding sites in the potential GAPDHS promoter. Active promoters are marked by high H3K4me3 enrichment and variable enrichment levels of H3K27ac (Marathe et al., 2017) and general enrichment of CG islands; we found high levels of H3K4me3 and enrichment of CG islands around the first exon of GAPDHS according to the UCSC database (Figure 7D). Hence, a ChIP-PCR assay was performed to verify our hypothesis. As shown in Figure 7E, we showed that SOX10 was enriched at the potential promoter of GAPDHS in OCM1 and OM431 cells. Thus, these data demonstrate that SOX10 can serve as a transcriptional factor to bind to the promoter of GAPDHS to regulate its expression at the transcriptional level and indicate that the potential binding site may be around the first exon.

In conclusion, our study demonstrates the essential role of SOX10 and GAPDHS in melanoma glycolysis and highlights their potential as therapeutic targets in melanoma management. SOX10 knockdown reduced the activation of GAPDHS, leading to an attenuated malignant phenotype, while SOX10 overexpression promoted the activation of GAPDHS, leading to an enhanced malignant phenotype. Furthermore, SOX10 exerts its function through GAPDHS by binding to its promoter to regulate its expression, which modulates glycolysis to ultimately affect the tumorigenesis of UM.

## Discussion

Glyceraldehyde-3-phosphate dehydrogenase not only plays vital roles in the glycolytic and gluconeogenic pathways but also relates to various non-glycolytic activities (Demarse et al., 2009). GAPDH may play a role in the neurodegenerative disorders such as Alzheimer’s disease and Huntington’s disease and might participate in both proapoptotic and oncogenic processes (Seidler, 2013). GAPDHS may be a substitute for GAPDH because it has high sequence identity and mainly plays roles in glucose metabolism, converting glyceraldehyde-3-phosphate to 1,3-bisphosphoglycerate. This protein has unusual properties different from its somatic isoenzymes and could significantly influence metabolism and proliferation (Sevostyanova et al., 2012), and the presence of GAPDHS confers a number of unusual properties compared with those of GAPDH in melanoma.

First, while GAPDH is not significantly differentially expressed in UM compared with normal controls and is commonly used as a control for gene expression studies, GAPDHS is specifically expressed in melanoma and in sperm and is expressed at low levels or not expressed in other tumors according to the GEPIA database, and it regulates tumorigenesis by modulating glycolysis efficiently to control glycolytic flux, which suggests that GAPDHS may play a more important role than GAPDH and that inhibitors targeting GAPDHS may be an approach with therapeutic benefit in melanoma.

Second, GAPDHS is known to exhibit higher stability than GAPDH (Elkina et al., 2010), which may cause the enzyme to hinder the translocation of GAPDHS into the nucleus to induce cell apoptosis that may play a role in the malignant phenotype of melanoma cells (Sevostyanova et al., 2012). In addition, the GAPDHS sequence does not possess a nuclear export signal (Kuravsky and Muronetz, 2007) and consequently cannot be involved in the induction of apoptosis. In our study, we found that there was no significant difference in cellular apoptosis after GAPDHS knockdown (data not shown). Therefore, GAPDHS may possess an important function for which GAPDH cannot compensate that leads to the high malignant potential of UM.

Finally, recent studies have shown that some glycolytic enzymes are complex, multifunctional proteins rather than involved in the glycolytic pathway (Kim and Dang, 2005), and they have been observed in the nucleus where they participate in tumor development independent of their non-classical metabolic roles; these multifaceted glycolytic enzymes are involved in various subcellular functions, suggesting that they not only play a role in classical metabolism but also directly link metabolism to epigenetic and transcription programs involved in tumorigenesis (Kim and Dang, 2005; Ganapathy-Kanniappan and Geschwind, 2013; Yu and Li, 2017). New functions of GAPDH, including transcriptional regulation and apoptosis, have been demonstrated with its nuclear localization; it also functions in processes such as membrane fusion, phosphorylation, tubulin bundling, nuclear RNA export, DNA repair, and interaction with cellular components, including RNA, dinucleoside polyphosphate, and nitric oxide (Kim and Dang, 2005). Regarding the nuclear functions of GAPDH, it has been reported that GAPDH might be involved in both proapoptotic and carcinogenesis processes (Seidler, 2013). Its carcinogenesis role involves the indirect participation in nucleic acid binding properties of hepatitis viruses – a function that correlates with liver carcinogenesis (Ganapathy-Kanniappan et al., 2012) – and the dissociation of GAPDH will cause the protein to be transported to the nucleus (Arutyunova et al., 2003) to play its proapoptotic role. Studies have demonstrated that glycolytic enzymes translocate to different subcellular compartments where they can interact with subcellular structures to an appreciable degree, resulting in significant differences in their primary and secondary functions. Furthermore, increasing evidence has shown that the combined use of small molecules to target both metabolic enzymes and their other non-metabolic functions could amplify their effects (Yu and Li, 2017). It would be reasonable to further study whether GAPDHS can translocate into the nucleus and exert its function as a nuclear protein, such as the nuclear forms of HK2, PKM2, LDH, and GAPDH. In summary, our study demonstrates the essential role of GAPDHS in melanoma glycolysis and highlights its potential as a therapeutic target in melanoma management.

In our study, we initially found that elevated SOX10 can affect glucose consumption, lactate production and ATP levels and consequently promote melanoma cell proliferation. We observed that GAPDHS is positively correlated with SOX10 expression; that is, GAPDHS expression increased when SOX10 was overexpressed, and vice versa. The transcriptional activity of SOX proteins differs dramatically, and some only exhibit transcriptional activity in combination with other transcription factors (Kuhlbrodt et al., 1998). Thus, it was interesting to analyze how SOX10 behaves as a transcription factor. We further verified by ChIP assay that SOX10 activates GAPDHS expression by binding to the promoter region. However, regarding the mechanism of GAPDHS expression, there are questions to be addressed: on the one hand, SOX proteins generally require other transcription factors as partner proteins to affect DNA topology and to shape the conformation of enhancer-bound multiprotein complexes as architectural proteins. For instance, SOX10 can interact with a diverse set of classical transcriptional regulators, such as Pax-3, CREB, and eventually Mitf (Wegner, 2005), to affect gene expression. We confirmed that SOX10 modulates GAPDHS expression; however, the possible key mediators involved in mediating the direct or indirect binding of SOX10 to the promoter of GAPDHS need to be further studied. Moreover, GAPDHS was identified as one of the top differentially expressed genes in human melanoma cells with NGLY1 knockdown (Zolekar et al., 2018), so it is also important to determine whether SOX10 cooperates with NGLY1 or other transcriptional regulators to promote melanomagenesis. On the other hand, as characterization of SOX10 binding sequences helps to identify those genes that are directly targeted by SOX10 and can further elucidate the mechanisms of SOX10-mediated gene activation, we screened the genomic region of the GAPDHS transcriptional unit for putative SOX10 binding sites and identified three putative SOX10 binding sites that were also present in the putative promoter region of GAPDHS. Thus, combined with H3K4me3 and CpG island enrichment, it is reasonable to determine the sequence around the first exon as the promoter, and it is noteworthy to investigate whether SOX10 directly binds the identified target sites within this promoter region with high affinity or cooperates with other transcription factors via direct protein–protein interactions, as with SOX10–PAX3 synergism (Mollaaghababa and Pavan, 2003).

## Data Availability Statement

The original contributions presented in the study are included in the article/Supplementary Material, further inquiries can be directed to the corresponding authors.

## Ethics Statement

The animal study was reviewed and approved by the Ethics Committee of Shanghai Ninth People’s Hospital.

## Author Contributions

XD, LW, and MC performed the experiments and drafted the manuscript. YW and MC were responsible for the data analysis. SG and JL discussed and revised the manuscript. XF and ML designed the study, wrote and approved the manuscript. All authors approved this submitted manuscript.

## Conflict of Interest

The authors declare that the research was conducted in the absence of any commercial or financial relationships that could be construed as a potential conflict of interest.

## Supplementary Material

Supplementary Figure 1 Flow cytometry was used to measure the change in the cell cycle of OCM1 cells with GAPDHS loss-of-function with two shRNAs. NTC indicates cells expressing non-targeting control. shGAPDHS-1 and shGAPDHS-2 indicates cells harboring shRNAs of GAPDHS. **P* < 0.05.
