# Supplementary figures and images for: Sperm-Specific Glycolysis Enzyme Glyceraldehyde-3-Phosphate Dehydrogenase Regulated by Transcription Factor SOX10 to Promote Uveal Melanoma Tumorigenesis
Source: Front Cell Dev Biol. 2021 Jun 25;9:610683. doi: 10.3389/fcell.2021.610683 (PMC8267526; doi:10.3389/fcell.2021.610683)

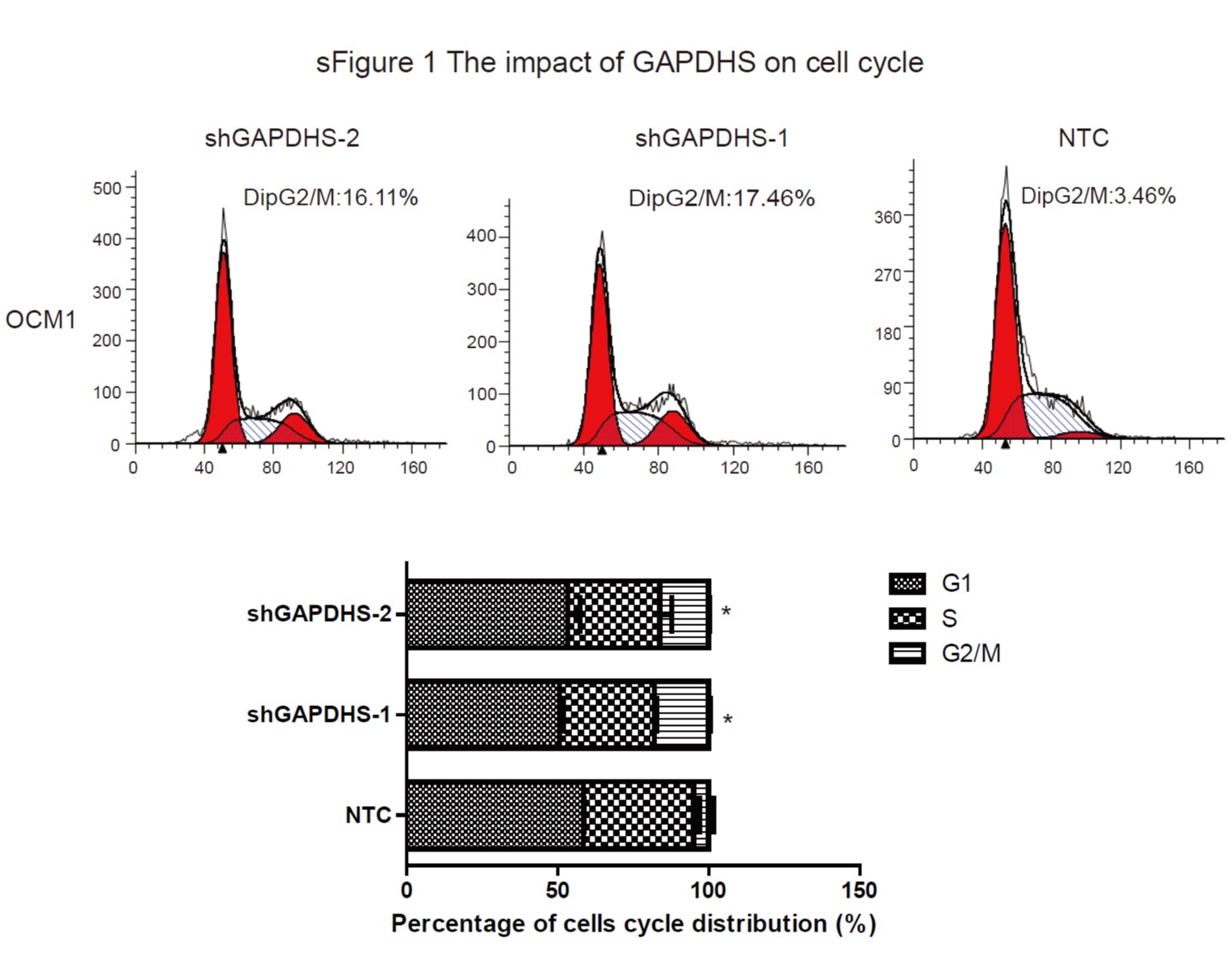

Supplement: Supplementary Figure 1 — Flow cytometry was used to measure the change in the cell cycle of OCM1 cells with GAPDHS loss-of-function with two shRNAs. NTC indicates cells expressing non-targeting control. shGAPDHS-1 and shGAPDHS-2 indicates cells harboring shRNAs of GAPDHS. *P < 0.05. [file Image_1.jpg]
